# Single-Cell Proteomics Reveals Specific Cellular Subtypes in Cardiomyocytes Derived From Human iPSCs and Adult Hearts

**DOI:** 10.1016/j.mcpro.2025.100910

**Published:** 2025-01-22

**Authors:** Lizhuo Ai, Aleksandra Binek, Vladimir Zhemkov, Jae Hyung Cho, Ali Haghani, Simion Kreimer, Edo Israely, Madelyn Arzt, Blandine Chazarin, Niveda Sundararaman, Jesse G. Meyer, Arun Sharma, Eduardo Marbán, Clive N. Svendsen, Jennifer E. Van Eyk

**Affiliations:** 1 Smidt Heart Institute, Cedars-Sinai Medical Center, Los Angeles, California, USA; 2 Advanced Clinical Biosystems Research Institute, Cedars-Sinai Medical Center, Los Angeles, California, USA; 3 Department of Biomedical Sciences, Cedars-Sinai Medical Center, Los Angeles, California, USA; 4 Board of Governors Regenerative Medicine Institute, Cedars-Sinai Medical Center, Los Angeles, California, USA; 5 Department of Medicine Research, Cedars-Sinai Medical Center, Los Angeles, California, USA; 6 Cancer Institute, Cedars-Sinai Medical Center, Los Angeles, California, USA; 7 Department of Computational Biomedicine, Cedars-Sinai Medical Center, Los Angeles, California, USA

**Keywords:** single-cell analysis, cardiomyocytes, iPSCs, stem biology, heart biology

## Abstract

Single-cell proteomics was performed on human induced pluripotent stem cells (iPSCs), iPSC-derived cardiomyocytes, and adult cardiomyocytes. More than 700 proteins could be simultaneously measured in each cell revealing unique subpopulations. A subset of iPSCs expressed higher levels of Lin28a and Tra-1-60 towards the outer edge of cell colonies. In the cardiomyocytes, two distinct populations were found that exhibited complementary metabolic profiles. Cardiomyocytes from iPSCs showed a glycolysis profile while adult cardiomyocytes were enriched in proteins involved with fatty acid metabolism. Interestingly, rare single cells also co-expressed markers of both cardiac and neuronal lineages, suggesting there may be a novel hybrid cell type in the human heart.

## Highlights

• One of the largest and most diverse single-cell proteomics datasets across five differentiation stages from iPSCs to iCMs and adult CMs.
• Large-scale single-cell proteomics approach captures major cell subpopulations in complex cell mixtures.
• Single-cell proteomics uncover rare cells comprised of hybrid proteomes of canonical cardiac-specific proteins alongside neuronal-enriched proteins in both aCMs and iCMs.
• Differentiated iCMs differ both in levels of structural proteins involved in muscle contraction and metabolic pathways.
• Similarly, Day 21 iCMs cluster into two subgroups separated at the level of metabolic proteins such as glycolysis, and myofibril proteins.

## Graphical Abstract

**Figure:**
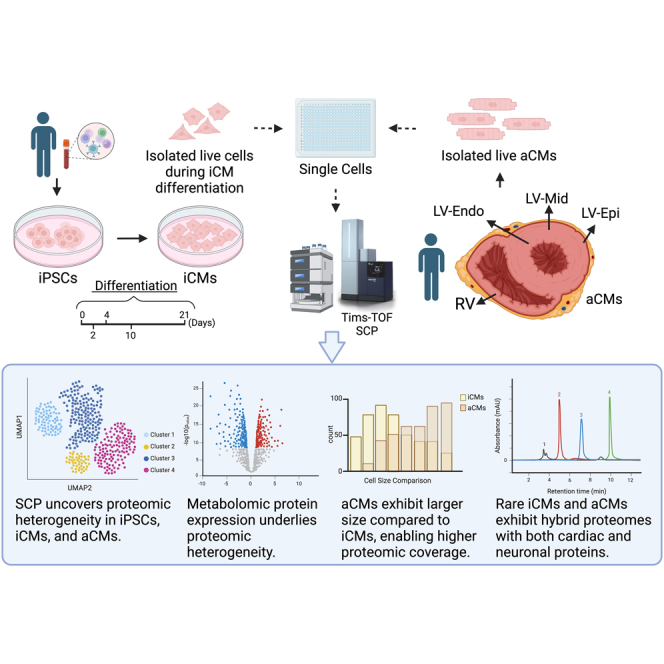

## In Brief

Ai *et al*. report single-cell proteomics characterization of the differentiation trajectory of cardiomyocytes derived from induced pluripotent stem cells and cross-compare them to adult cardiomyocytes from the human heart. The single-cell proteomic approach captures complex cell state heterogeneity and reveals distinct cardiomyocyte subtypes characterized by metabolic and contractile signatures.

Induced pluripotent stem cell (iPSC)-derived human cardiomyocytes (iCMs) are proposed as tools to model cardiovascular diseases and drug toxicology but this requires that they recapitulate some features of cardiomyocytes from adult hearts (aCMs) (1, 2). Therefore, like aCMs, differentiated human iCMs would need to (i) express specific adult myofilament protein isoforms and (ii) have undergone a metabolic aerobic protein shift to encompass glucose and fatty acid metabolism. Mass spectrometry (MS)-based proteomics at the single-cell resolution is an emerging technique to characterize the heterogeneity of cell states (3). However, current technical bottlenecks include slow single-cell processing time and low-throughput and/or the need for the application of labeling MS technologies (4, 5, 6, 7, 8).

Based on technological advances in single-cell MS-based proteomics, we have applied a simplified “one pot” label-free single-cell proteomic approach combined with our parallelized nanoflow dual-trap single-column liquid chromatography (9) in order to map proteomic changes during differentiation of iPSCs into iCMs, determine cell type and state flexibility and heterogeneity during maturation and, importantly, compare the proteomes of iCMs to that of freshly isolated aCMs from different regions of the human heart.

Our study shows that large-scale high-throughput single-cell proteomics can be used to reliably detect proteins and demonstrate a new proteomic map of both iCMs and aCMs that can be used to understand pluripotency, cardiovascular diseases, and drug toxicology. Furthermore, aCMs were isolated from various parts of the heart that are known to have different functions and contractile properties (10, 11, 12) to determine whether iCMs represent any specific anatomical region or a generic CM phenotype.

## Experimental Procedures

### iPSC Culture and iCM Differentiation

The CS0202iCTR human iPSC line was generated by the Cedars-Sinai Medical Center iPSC Core from peripheral blood mononuclear cells of a healthy male individual with nonintegrating oriP/EBNA1 plasmids, which allowed for episomal expression of reprogramming factors and shown to be fully pluripotent (13, 14). iPSCs were maintained in mTESR1 medium on Matrigel-coated cell culture plates and passaged every 5 days at split ratios from 1:6 to 1:12 as needed using Versene. Only iPSCs between passage 17 and passage 35 were used for differentiation in this study (13).

The iPSCs were differentiated into cardiomyocytes (iCMs) using an established monolayer differentiation protocol utilizing small molecule modulators of Wnt signaling (14). Differentiated iCMs were metabolically purified by depriving cells of glucose, as previously demonstrated (14). Purified iCMs expressed standard cardiac sarcomeric markers cardiac troponin T (cTnT) and α-actinin.

### iPSCs to iCMs Isolation and Sorting

At each time point, cells were dissociated with Accutase, collected, and resuspended in PBS + 0.5 mM EDTA buffer. Cells were stained for viability with Sytox Green dye (Thermo Scientific S7020, 1:5000) for 30 min on ice, washed in PBS + 0.5 mM EDTA buffer, and dispensed using FACS-sorting machine (BD Biosciences) cell sorter, using a 100 uM nozzle and the “1.0-drop Single” sort setting with a 12/16 phase mask into separate wells on a 384-well low binding PCR plate (Biorad HSP3801) containing 200 nl of lysis buffer (100 mM TEAB, 0.2% DDM, 10 ng/nl trypsin). Each experiment contained two rows of cells with 10 cells and 50 cells which were used as reference for library preparation. Plates were covered with foil and stored at −80 °C for further processing.

### Human Heart Transportation and aCMs Isolation and Sorting

The hearts were screened by transthoracic echocardiogram, and only the hearts with normal systolic function (ejection fraction >40%) and diastolic function (normal E/A ratio and E/e’ ratio) were included (Table S2). Single cardiomyocytes were isolated from the chunks of the left ventricular free wall (transported in University of Washington solution) using enzymatic digestion isolation technique (in calcium-free Tyrode solution with collagenase II [1.0 mg/ml, Worthington Biochemical Corporation] and protease XXIV [0.1 mg/ml, Sigma-Aldrich]) in a swirling flask for 60 to 90 min. Ventricular single cells were prepared after filtration with a 200 μm filter and centrifugation to remove debris. Individual aCMs were dispensed into separate wells on the same type of Bio-rad 384-well plate containing the same lysis buffer as for iCMs as the using a CellenONE (Cellenion) as previously reported (15). To limit a potential bias during sample preparation, all samples were provided a coded biospecimen name throughout the entire process. During sample preparation and mass spectrometry analysis, all personnel involved were blinded to the human subject information and biospecimen coding except for one scientist who carried out the plate mapping to ensure randomization of samples but who was not involved in data acquisition. Once the data acquisition and raw data file pre-processing using the coded information was complete, one data analyst was provided the de-identified information to proceed with the downstream bioinformatics processing.

### Sample Preparation for MS

After lysis by freeze-thaw, all samples (single or pooled iCMs and aCMs) were subjected to trypsinization with 40 ng/μl trypsin, 4 h incubation at 37 °C, followed by acidification with 0.1% formic acid. All five timepoint 384-well plates were digested in the same batch and all three human plates were digested in the same batch before proteomic analysis using Tims-TOF single-cell proteomics.

### DIA MS Proteomic Analysis

The high throughput liquid chromatography set up for single-cell analysis has been previously developed by our group which involves the nanoflow dual-trap single-column configuration (nanoDTSC) (9). This configuration capitalized on parallelized nanoDTSC chromatography operating at 10 min of total run time per cell with peptides quantified over 8 min, thus offering an efficient solution to injection of 144 wells per day. Key modifications to the previously published article included integrating a 5 cm × 75 μm ID Ionopticks analytical column packed with 1.5 μm C18 material and interfacing it with the Bruker TimsTOF single-cell proteomics mass spectrometer. These enhancements significantly improve throughput and sensitivity, thereby facilitating comprehensive single-cell proteomic analysis.

The analytical separation protocol entails using 0.1% formic acid in water as mobile phase A and 0.1% formic acid in acetonitrile as mobile phase B. The gradient profile begins at 9% B with a flow rate of 800 nl/min, gradually increasing to 20% B over 5 min, then to 40% B over 3.9 min. Subsequently, the flow rate ramps up to 1400 nl/min, reaching 98% B within 0.1 min, maintained for 0.5 min, followed by a drop to 9% B at 1200 nl/min over 0.05 min. The system holds at 8% B for 0.25 min before dropping the flow rate to 800 nl/min for 1 min, resulting in a total run time of 10 min.

The loading pump initiates the run by delivering mobile phase A (100% 0.1% formic acid in water) at 100 μl/min for the first 0.5 min, then gradually increasing the flow to 120 μl/min over 0.3 min, maintained for 5 min before dropping to 8 μl/min until the end of the 10-min run. The valves and trapping columns are regulated at 55 °C in the Ultimate 3000 column oven compartment, while the analytical column is maintained at 60 °C using the Bruker “Toaster” oven.

In this setup, the analytical column is directly linked to a 10 μm ZDV emitter (Bruker) installed in the Bruker captive source, interfacing with the Bruker’s TimsTOF single-cell proteomics mass spectrometer. The capillary voltage is set to 1900V, with dry gas flowing at 5.0 L/min and a temperature of 180 °C. Data acquisition employs DIA-PASEF, with ion accumulation and trapped ion mobility ramps set to 166 ms. DIA scans cover 90 m/z windows spanning 300 to 1200 m/z and 0.6 to 1.43 1/K0, with one full MS1 scan followed by four trapped ion mobility ramps, resulting in a cycle time of 0.86s.

### Proteomics Data Analysis

Data were analyzed with DIA-NN 1.8.1 (16, 17) and DIA-NN 1.8.2 (beta 27) using sample-specific libraries for each dataset: iPSCs to iCMs differentiation time course and human primary aCMs, respectively. Uniprot FASTA database from Aug 30th 2023 containing 20,423 protein entries (Swiss-Prot reviewed only) was used in the data processing steps. No fixed modifications and 1 variable modification (Unimod:35 - Methionine oxidation), minimum peptide length-7 amino acids, and maximum peptide length-40 amino acids; were set during the generation of the spectral library. Each search was conducted with second-pass and match-between-runs (MBR) enabled. The mass error tolerances were set at 15 ppm for both fragment and intact masses.

For analyzing the iPSCs and iCMs differentiation samples, the 50-cell samples DIA runs with the highest identifications were analyzed using the library-free search in DIA-NN against the complete human protein UniProt database to create a spectral library. The library-free identifications are set at <1% false discovery rate (FDR) to generate the final library by DIA-NN using the target-decoy strategy used for the analysis of iCMs cell lines.

For analyzing the aCMs from the 3 humans, an initial DIA library was generated using the 20 highest performing single aCMs in DIA-NN. The library was next used for analysis of aCMs from all three humans. The identification false discovery rate in cell analysis was filtered to 1% by DIA-NN using the target-decoy strategy.

For both iCMs and aCMs data processing and visualization, protein level quantitation was used to cluster individual cells using the Python Single-Cell Analysis package (SCANPY) (18), which models the data as a connected graph. Protein abundance was log-transformed. Protein abundance was normalized to the total count in the two human samples. No imputation method was used. A filtering criterion was applied to the datasets, specifying a minimum of 10 proteins and a maximum of 1600 proteins per cell, with each protein being detected in at least 10 cells. Dimensionality reduction was performed using Uniform Manifold Approximation and Projection (UMAP) (19) embedding. For this paper, we used 15 neighbors to compute local connectivity, excluded cells with proteins outside of two standard deviations and used only the presence of proteins to determine their relevance. We did not consider the absence of proteins in the determination of relevance. Trajectory analysis was performed using Monocle 3 (20). Cell cycle analysis was performed using a set of previously annotated cell cycle markers (21).

### Immunocytochemistry Analysis

For immunocytochemistry of Day-0 iPSCs, colonies were grown on glass coverslips coated with Matrigel (Lab-Tek II, Thermo Scientific) and then fixed with 4% paraformaldehyde. Cells were blocked and permeabilized in 5% BSA +0.5% Triton X-100 in PBS and stained overnight with the antibody against Lin28a (1:1000, MA1-016, Thermo Scientific). The next day, samples were washed and stained with an Alexa594-conjugated anti-mouse antibody, counterstained with DAPI, and imaged using a confocal microscope.

### Experimental Design and Statistical Rationale

Biological sample from each iPSC differentiation time point underwent single-cell proteomics analysis 288 times, 10 pooled single cells 48 times, and 50 pooled single cells 48 times with dual trap nanoLC mass spectrometry method while maintaining consistent chromatography conditions monitored by periodic HeLA QC measurements (see Supplemental Fig. S13). The order of differentiation time points sample processing and MS acquisition was performed in a randomized fashion. This work resulted in a total of 1920 LC-MS raw data acquisitions subjected to peptide identification by DIA-NN software.

All human hearts explants were obtained from the National Disease Research Interchange (Philadelphia, PA) under the IRB-approved Cardiovascular & Thoracic (Cardiothoracic) Tissue Repository (Pro00010979 approved in 2007.) at Cedar-Sinai Medical Center. The human studies reported in this manuscript abide by the Declaration of Helsinki principles. Only a limited number of human heart biospecimens was able to be procured (n = 3) and yielded a total of n = 159, n = 128, and n = 23 LC-MS raw data files capturing single-cell proteomic measurements, respectively for Human 1, 2, and 3.

Cell populations with similar protein compositions were grouped into clusters using Leiden clustering. To assess the statistical significance of protein changes between different Leiden clusters (0, 1, and 2) at 21 days, an unpaired two-sample *t* test was employed (*p* value < 0.05). In protein ranking tests to determine the topmost (top 5) expressed proteins in different Leiden clusters (0, 1, and 2) at 21 days Benjamini-Hochberg post-correction test was applied (aj. *p* value < 0.05).

## Results

**Fig. 1:**
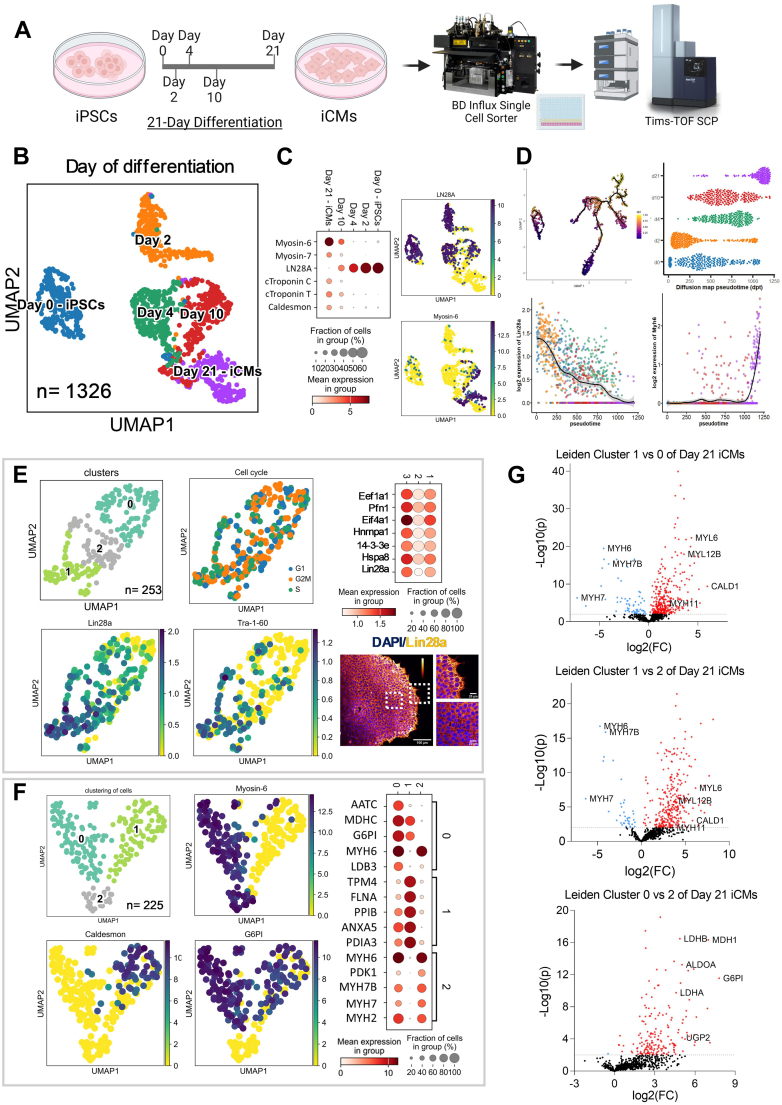
**Single-cell proteome trajectory of differentiating cardiomyocytes.***A*, workflow from iCM differentiation to single-cell proteomics acquisition. *B*, UMAP of the proteome of single cells at each differentiation time point. *C*, dot plot of cardiomyocyte marker proteins expressed at each time point; UMAPs of LN28A (stem cell marker) and MHY6 (cardiomyocyte marker). *D*, Pseudotime trajectory of iCM differentiation and expression of LN28A and MHY6 at the single-cell level. *E*, UMAP of only Day-0 iPSCs identified in 3 Leiden clusters; UMAPs colored by markers of cell cycle stage, LN28A, and Tra-1-60; Dot plot represents the top seven proteins differentially expressed in subcluster 1 of iPSCs; Immunocytochemistry analysis of Lin28a expression in iPSC colonies at Day0. *F*, UMAPs of only Day-21 iCMs identified in 3 Leiden clusters; Dot plot top 5 proteins expressed in each cluster. *G*, Volcano plots of Day-21 iCMs cluster 1 vs cluster 0, cluster 1 vs cluster 2, and cluster 0 vs cluster 2. (enriched proteins are highlighted in *red*; depleted proteins are in *blue*). The color bar labels in all UMAP figures represent log fold change values of protein intensity.

iPSCs were differentiated into iCMs using a combination of small molecule morphogens (days 0–10) and further purified using metabolic selection by glucose starvation (days 10–15) as described previously (14, 22). Individual cells were collected on Day0 (iPSC), Day2 (mesoderm), Day4 (cardiac progenitor), Day10 (non-purified contracting iCMs), and Day21 (differentiated purified contracting iCMs) (Fig. 1*A*). Cells at each timepoint were dissociated into single-cell suspension, labeled with a viability dye (Sytox Green dye) and isolated by fluorescence-activated cell sorting to single wells (n = 288 single cells) of a 384-well plate containing pre-dispensed lysis buffer (9) (Fig. 1*A*). Each timepoint also included 10-cell and 50-cell samples used in data-independent acquisition-MS (DIA-MS) as the spectral library build. Prototypic peptides (mapped exclusively to a single protein) identified at 1% FDR were merged, filtered, and log-transformed across cells at all time points. Consistent proteome coverage was observed among all samples in five differentiation time points (Supplemental Fig. S1). When all time points were combined, 2742 unique proteins were identified from a total 1326 single cells that underwent single-cell proteomics analysis (see Supplemental Fig. S13 for dataset’s and its associated QC samples’ general characteristics, including missingness, missed cleavages, protein, and peptides %CV).

Single-cell proteome changes in all five differentiation time points were visualized by performing dimensionality reduction on the UMAP plots (Fig. 1*B*). Unbiased Leiden clustering, which identifies communities or clusters of cells based on their connectivity (23), showed that single-cell proteomes were driven by timepoint differences and were independent of total protein count per cell, indicating the cell proteome was distinct at each differentiation timepoint (Supplemental Fig. S2). The stem cell marker Lin28a decreased over time, while canonical cardiomyocyte markers (e.g., Myosin-7 (MYH7) and Myosin-6 (MYH6)) gradually increased over time and were evident in a subpopulation of iCM cells at Day21 (Fig. 1*C*). Pseudotime analysis of the single cell proteome trajectory from Day0 iPSC to Day21 indicated a maturation to cardiomyocytes through the sequential timepoints (Fig. 1*D*). But, it also revealed rare individual cells that expressed canonical stemness and cardiac-specific proteins along the trajectory, suggesting some flexibility of proteome expression in single cells using this differentiation protocol (Fig. 1*C*).

Interestingly, there was also within-cluster heterogeneity at each time point. Sub-clustering of the iPSCs at Day0 showed that a proportion of the cells (cluster 1) was characterized by higher expression of pluripotent stem cell markers (Lin28a, Tra-1-60) and proteins related to RNA metabolism and transcription (Fig. 1*E*). Higher expression was independent of cell cycle stage and represents an interesting subset of pluripotent cells based on protein expression previously not seen with single-cell RNA sequencing (scRNA-seq) profiling (24). Antibody staining of growing iPSCs for Lin28a revealed a small population of highly expressing cells at the edge of cell colonies (Fig. 1*E*), supporting the existence of a subset of pluripotent cells with distinct protein expression profiles within the iPSC population.

At Day21, the majority of iCMs were enriched for cardiac-specific markers (e.g., MYH6, cardiac adult isoform troponin T (cTnT)), although there was also a small portion of the cells with very limited cardiac-specific proteins present (Fig. 1*C* and Supplemental Fig. S3). Based on Leiden clustering, three separate sub-populations of cells were found on Day21 (n_cluster0_ = 107 cells; n_cluster1_ = 90 cells; n_cluster2_ = 28 cells; Fig. 1*F* and Supplemental Fig. S4*A*). The top 5 proteins expressed in each cluster indicated that MYH6 was highly expressed in both cluster 0 (101/107 cells) and cluster 2 (25/28 cells), but minimally expressed in cluster 1 (6/90 cells; Fig. 1*F*), suggestive that clusters 0 and 2 correspond to iCMs. Furthermore, volcano plots of the differentially expressed proteins revealed that clusters 0 and 2 expressed cardiac-specific proteins whereas cluster 1 had significantly higher protein markers of smooth muscle cells, including Caldesmon (CALD1), Myosin-11 (MYH11), Myosin light chain 6 (MYL6), and Myosin light chain 12B (MYL12B) (Fig. 1*G*).

When focusing on iCMs (Cluster 0 and 2) a cardiac-specific adult isoform of myofilament proteins (MYH6 and cTnT) was detected. Interestingly, metabolism (represented by 101 proteins) was an important driver for the differences between Clusters 0 and 2 comprising iCM cells, specifically glycolysis (G6PI: Glucose-6-Phosphate Isomerase, LDHA: L-lactate dehydrogenase A chain, LDHB: L-lactate dehydrogenase B chain, MDH1: cytoplasmic Malate dehydrogenase, ALDOA: Fructose-bisphosphate aldolase A, UGP2: UTP--glucose-1-phosphate uridylyltransferase), although both clusters expressed similar intensity of canonical cardiomyocyte protein markers (Supplemental Table S1*A*, Fig. 1, *F* and *G*). Glycolysis is the main energy source for immature cardiomyocytes (25, 26), thus the enrichment of glycolytic proteins in cluster 0 suggested an array of proteomic maturity in this subset of the Day21 iCMs. In addition, within cellular component gene ontology (GO) terms from the ClueGO analysis (27) between clusters 0 and 2 (Supplemental Table S1*B*), 185 vesicle proteins (of 178 involved in extracellular exosomes) were upregulated in Cluster 0 iCMs. Extracellular exosomes isolated from iCMs culture media have been suggested to hold great therapeutic potential for treating myocardial injuries (28, 29) and our results are the first to imply iCMs heterogeneity in producing these vesicles, providing novel insights into the diversity of iCMs and their potential roles in therapeutic applications.

To further define the Day21 iCMs, representative cardiomyocyte (e.g., MYH6), smooth muscle cell (e.g., CALD1) and metabolic (e.g., G6PI) protein markers were plotted onto UMAP projections (Fig. 1*F* and Supplemental Fig. S4), which clearly stratified cells at Day21 into smooth muscle and two unique types of iCMs. Furthermore, UMAP visualizing CALD1 showed minimal overlap with cells expressing this smooth muscle marker with CMs (5/107 cluster 0 cells, 57/90 cluster 1 cells, and 0/28 cluster 2 cells at Day21; Fig. 1*F* and Supplemental Fig. S3*G*), supportive of a distinctive smooth muscle population. Yet, due to non-exclusivity, this also may indicate a set of rare hybrid cells that express proteomes comprised of both cell types. Furthermore, we found neuronal-specific proteins (*e.g*.: Enolase 2 – ENO2 and Neuroplastin – NPTN) also expressed alongside cardiac-specific proteins in a few isolated cells which may represent rare hybrid cells composed of a flexible cell proteome state (Supplemental Figs. S5 and S15). These proteomics results are supportive of previous scRNA-seq studies which had suggested iCMs heterogeneity (30) but better define the individual cell flexibility of expression.

**Fig. 2:**
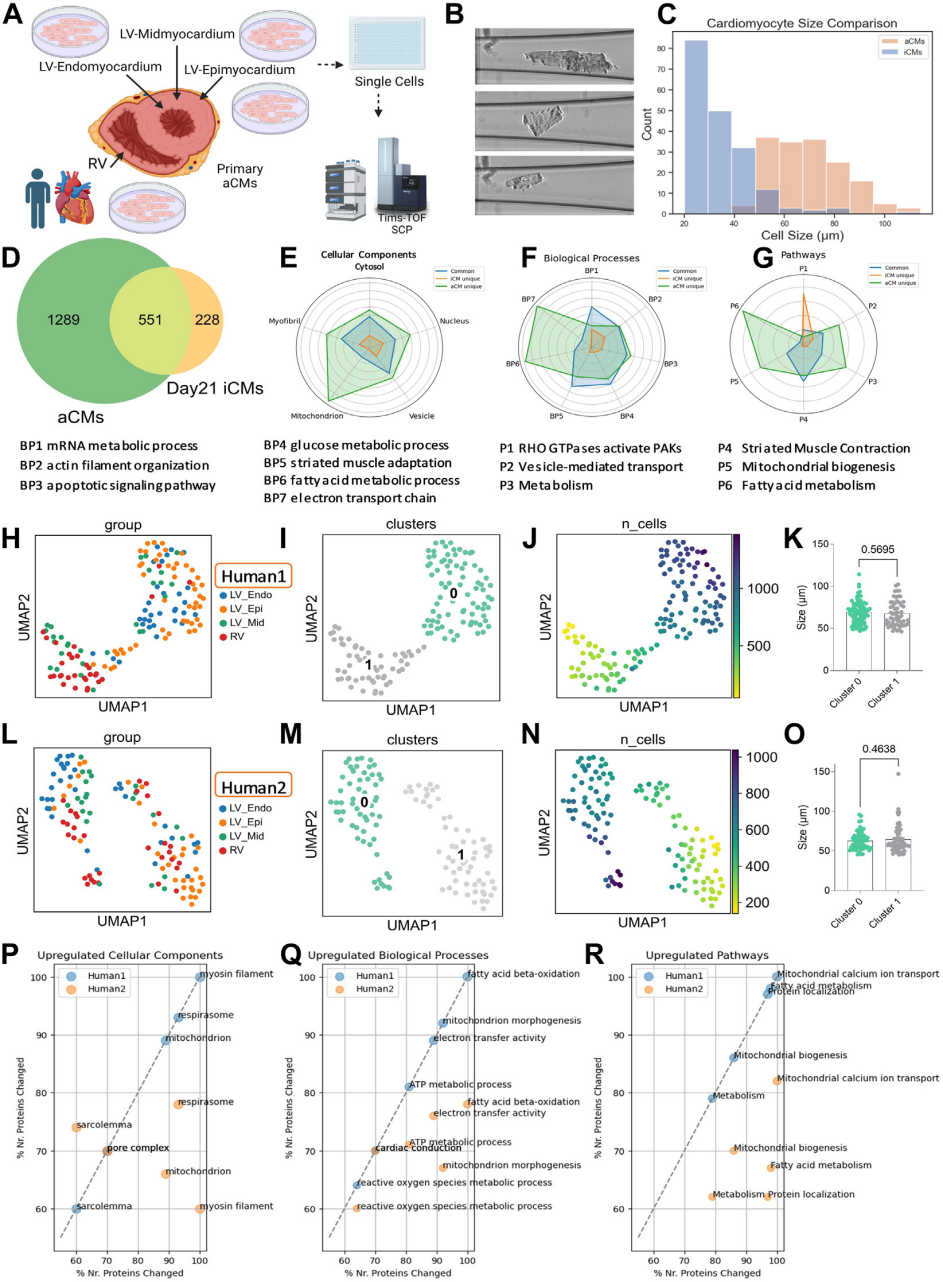
**Single-cell proteomics of aCMs.***A*, workflow of aCM isolation to single cell proteomics. *B*, representative images of aCMs in the dispensing capillary before sorting. *C*, Cell size comparison between aCM and iCM (aCMs=161 cells; iCMs=187 cells). *D*, proteome overlaps between aCMs and Day21 iCMs. *E*–*G*, radar plots comparing ClueGO enrichments in cellular components (*E*), biological processes (*F*), and pathways, in aCMs unique proteins (*green*), iCM unique proteins (*orange*), and the common aCM and iCM overlapping proteins (*blue*). *H*–*J*. UMAPs of individual aCM proteomes colored by the location isolated (*H*), two Leiden clusters (*I*), and protein count (*J*), in human1. *K*, cell size comparison between Leiden clusters 0 and 1 human1 aCMs. L-M. UMAPs of individual aCM proteomes colored by the location isolated (*L*), two Leiden clusters (*M*), and protein count (*N*), in human2. *O*, cell size comparison between Leiden clusters 0 and 1 human2 aCMs; *p* > 0.05, not significant by *t* test. *P*–*R*. ClueGO protein enrichments of cluster0 aCMs in cellular components (*P*), biological processes (*Q*), and pathways (*R*), compared to cluster1 aCMs in human1 (*blue*) and human2 (*orange*), respectively.

To benchmark iCMs single-cell proteome heterogeneity and to understand cell state specificity and cell type heterogeneity of aCMs, we next isolated ventricular aCMs from 3 sections of left ventricle (LV); (i) mid-myocardium (LV mid), (ii) endo-myocardium (LV endo), (iii) epi-myocardium (LV epi), and right ventricle (RV) (9) from three donor hearts. Dissociated cells stained for viability as above were isolated using CellenONE sorter, and only viable rod-shaped cardiomyocytes were dispensed and analyzed using DIA-MS (9) (Fig. 2, *A* and *B*). Using strict sorting criteria (see methods) we were able to dissect, isolate, and analyze 141 and 122 viable rod-shaped CMs from Human1 and Human2, but only 16 from Human3 due to the deteriorating condition of this heart.

Next, to assess the extent to which Day21 iCMs recapitulate the aCMs proteome, we first compared their morphology and observed that aCMs are double in cell size than iCMs (aCMs = 69 ± 15 μm; iCMs = 34 ± 13 μm; Fig. 2*C*, Supplemental Fig. S14), resulting in higher amount of protein analyzed per cell on the MS and which likely have contributed to the greater proteome coverage in aCMs (Fig. 2*D*, Supplemental Tables S3 and S4). Although there was a large degree of similarity with two-thirds of Day21 iCMs proteome overlapping with aCMs, within the categories of primary cellular components, mitochondrial proteins are more abundant in aCMs (15% common, 3% uniquely found in iCMs, and 82% uniquely identified in aCMs; Fig. 2*E*). This pronounced enrichment suggests that the proportion of mitochondrial proteins is higher in aCMs relative to iCMs. Further analysis supported that several mitochondrial processes are significantly more represented in aCMs, specifically the fatty acid metabolic process, electron transport chain, and mitochondrial biogenesis (Fig. 2, *F* and *G*). In addition, we observed a significant overlap of proteins involved in striated muscle adaption and contraction between iCMs and aCMs. This implies that iCMs share similarities with aCMs, particularly in muscle function, although iCMs display less mitochondrial protein content per cell and thus potentially a less mature energy metabolism than aCMs.

As with the known functional differences (11, 12), there is documented transcriptomic heterogeneity of aCMs between various areas of the heart as well as within anatomical structures (11, 31, 32). However, UMAPs of aCM single-cell proteomes suggested 2 cell clusters in each human heart, driven by total protein count but independent of aCMs anatomical location or cell size (Fig. 2, *H*–*Q*; Supplemental Figs. S6 and S7). The latter may be a limitation of the proteome coverage at the single-cell level. Moreover, we found neuronal-specific proteins (*e.g*.: Enolase 2 – ENO2 and Neuroplastin – NPTN) expressed alongside cardiac-specific proteins also in aCMs, consistent findings of a small population of rare hybrid cells composed of a flexible cell proteome state in aCMs (Supplemental Figs. S5 and S15) as with iCMs (Supplemental Figs. S5 and S15).

Importantly, the UMAP protein abundance projections of myosin isoforms and cardiac troponins demonstrated even distributions across both clusters (Supplemental Fig. S9). Functional enrichments of differential proteins in cluster 0 cells compared to cluster 1 cells were largely consistent in both patients (Fig. 2, *P* and *Q*). Notably, in both human subjects, we observed that cluster 0 cells are more highly enriched in mitochondrial proteins and metabolic pathways than in cluster 1. This finding is particularly intriguing as we previously observed that iCMs have a less mature metabolism-related proteome. iCM clusters were distinguished by glycolysis and lacking in the aerobic metabolism categories (e.g: Oxidative phosphorylation, Fatty Acids oxidation) that are the principal drivers of differences in Leiden clusters 0vs1 in aCMs. We also compared cardiac troponin T (cTnT) which is present only in adult cardiomyocytes with slow skeletal TnT (ssTnT) which is expressed in immature cardiomyocytes *in vivo*. 97% of all aCMs isolated from human subjects expressed cTnT with ssTnT being quantified in only a subset of cells and at a significantly lower protein abundance per cell than the adult isoform (Supplemental Fig. S10).

Another means to assess the maturity of iCMs is to compare the highest-ranking cardiac-specific myofilament protein isoforms of the Day-21 iCMs with aCMs. MYH7, which is expressed solely in adult hearts (33, 34), was the dominant myosin isoform (Supplemental Fig. S11) in aCMs regardless of the cluster. MYH6 was the dominant isoform in the two clusters of iCMs. MYH6 is expressed in adult heart muscle but shows expression flexibility as it is found in the atrium and ventricles during cardiac development (35) suggestive of a degree of proteomic immaturity in iCMs compared to aCMs. Proteomic data also showed that iCMs are highly enriched in proteins in the pathway of Rho GTPases activating PAKs (Fig. 2*G*), and PAKs have been suggested to be important in heart development (36) and regulation of calcium handling in cardiomyocytes (37, 38). However, importantly, other developmental myofilament isoforms, such as Myosin heavy chain 3 (MYH3) (39, 40), were not detected in the iCM subclusters. These data suggest that, while immature, iCMs are progressing toward maturity.

Finally, both aCMs and iCMs revealed single-cell proteome heterogeneity both with respect to cell state and cell type. While the expression of canonical cardiac markers was evenly distributed across Leiden clusters both in iCMs and aCMs, we did observe an enrichment in myofibril contractile category for a subcluster of cells (specifically belonging to Leiden clusters 0) in either cell type (Supplemental Table S1 and Supplemental Fig. S12). Additionally, although fatty acid metabolism difference was observed between aCM clusters, likely due to the high proteomic coverage for the mitochondrial proteins (Fig. 2*E*), we observed cluster similarity in other metabolic processes when between aCMs and iCMs, including cellular amino acid metabolic process and carbohydrate metabolic process (Supplemental Fig. S12). Together this data suggests that, aside from the main mitochondrial protein differences between iCMs and aCMs, there are notable proteome similarities when performing sub-cluster comparisons in these cells.

## Discussion

In summary, several other groups have reported single-cell proteomic heterogeneity of various cell populations but were limited by time-per-sample analysis, complicated labeling techniques, sample size, or cell type diversity (4, 5, 6, 7, 8). In the current study, we applied a label-free single-cell proteomics approach, which is required due to the range in cell size in both iCMs and aCMs. We report one of the largest and most diverse datasets acquired to date by analyzing 1326 single cells across five different cell states derived from iPSCs and furthermore, we are the first to isolate live rod-shaped adult cardiomyocytes from multiple human heart tissues for single-cell proteomics studies. Using our approach, we were able to characterize proteomic composition within the starting iPSC population revealing interesting heterogeneity at the protein level which may allow a better characterization of the pluripotent state. As the cells progressed along a differentiation trajectory into cardiomyocytes the exact protein profile was established and distinct cellular subtypes within cell populations were identified. Consistent with earlier single-cell transcriptomic studies (30), we observed heterogeneity at Day21 iCMs manifested both in levels of structural proteins involved in muscle contraction as well as in metabolic pathways such as glycolysis. Like aCMs, iCMs at Day21 cluster into two subgroups of cardiomyocytes which were separated at the level of metabolic proteins such as glycolysis, and myofibril proteins. This is in line with the postnatal cardiomyocyte feature of maturation characterized by the metabolic switch from glycolysis to fatty acid β-oxidation (25, 41, 42) and the embryonic-to-adult switch in contractile myosin/TnT protein isoforms observed in both iCMs and aCMs. Finally, single-cell proteomics uncovered rare cells comprised of hybrid proteomes of canonical cardiac-specific proteins alongside neuronal-enriched proteins in both aCMs and iCMs. These rare hybrid cells may represent cell-type flexibility previously not seen.

Taken together, our approach shows for the first time the feasibility of large-scale high-throughput single-cell proteomics studies across a wide range of related cell types. Based on the single-cell proteomic changes during iPSC differentiation to iCMs and comparing their proteomes to that of isolated aCM led to the discovery of unexpected cell states with two subclusters of CMs and cell type heterogeneity with rare cells that co-express cardiac and other cell type-specific proteins, indicating the need for a more flexible view of the cardiomyocyte proteome.

## Data Availability

The presented iPSC to iCMs data is shared on MassIVE: MSV000094438, PXD051092, DOI: 10.25345/C5T727S7Q. To view the dataset's files (including title, description, and metadata), log in to the MassIVE FTP server with this URL: ftp://MSV000094438@massive.ucsd.edu, username: MSV000094438, password: iPSCs.

Human1 proteomic data has been previously published from our group (9) (MassIVE: MSV000090903, PXD038828, 10.25345/C5Z60C65X, password: dualtrapscp). Human2 and Human3 data were acquired using the same methods and now uploaded on MassIVE (MSV000094571, PXD051594, DOI:10.25345/C5MW28R7C, password: humancm).

## Supporting Information

This article contains supporting information.

## Conflict of Interest

The authors declare the following financial interests/personal relationships which may be considered as potential competing interests:

A collaborative agreement between Cedars-Sinai with Bruker Inc, to support analysis in biomarker development using a different instrument than used in this publication. Dr. Marbán has founder equity in Capricor Therapeutics. The authors declare no other competing interest.
