# Supplementary material for: Single-Cell Proteomics Reveals Specific Cellular Subtypes in Cardiomyocytes Derived From Human iPSCs and Adult Hearts
Source: Mol Cell Proteomics. 2025 Jan 22;24(9):100910. doi: 10.1016/j.mcpro.2025.100910 (PMC12445721; doi:10.1016/j.mcpro.2025.100910)
Supplement: Supplementary Data [file mmc1.docx]

**Supplemental Information**

Single Cell Proteomics Reveals Specific Cellular Subtypes in Cardiomyocytes Derived from Human iPSCs and Adult Hearts

Lizhuo Ai^1,2,3,4,^^, Aleksandra Binek^1,2,^,*^, Vladimir Zhemkov^4,^^, Jae Hyung Cho^1^, Ali Haghani^1,2^, Simion Kreimer^1,2^, Edo Israely^5^, Madelyn Arzt^1,3,4,6^, Blandine Chazarin^1,2^, Niveda Sundararaman^1,2^, Arun Sharma^1,3,4,6^, Eduardo Marbán^1,3, #^ Clive N. Svendsen^3,4,*,#^, Jennifer E. Van Eyk^1,2,3,*,#^

^1^ Smidt Heart Institute, Cedars-Sinai Medical Center, Los Angeles, CA 90048

^2^ Advanced Clinical Biosystems Research Institute, Cedars-Sinai Medical Center, Los Angeles, CA 90048

^3^ Department of Biomedical Sciences, Cedars-Sinai Medical Center, Los Angeles, CA 90048

^4^ Board of Governors Regenerative Medicine Institute, Cedars-Sinai Medical Center, Los Angeles, CA 90048

^5^ Department of Medicine Research, Cedars-Sinai Medical Center, Los Angeles, CA 90048

^6^ Cancer Institute, Cedars-Sinai Medical Center, Los Angeles, CA, 90048

*Corresponding authors: Clive.Svendsen@cshs.org & Jennifer.VanEyk@cshs.org & Aleksandra.Binek@cshs.org

^ Co-first authors who contributed equivalently

# Co-senior authors who contributed equivalently

**Contents**

**Supplementary Figures**

Figure S1. Protein coverage of the single cells at each differentiation stage.

Figure S2: Distinct proteomic profiles at each differentiation timepoint.

Figure S3. Cardiomyocyte marker expression increase with differentiation.

Figure S4: UMAPs of representative cardiomyocyte, metabolic and smooth muscle cell markers in Day-21 iCM cells.

Figure S5: UMAPs of representative cardiomyocyte or neuronal cell marker expression progression through iCM differentiation.

Figure S6. A. UMAP plot of aCMs from 3 human subjects combined colored by individual humans.

Figure S7. Proteome overlaps between cluster 0 cells and cluster 1 cells human individuals.

Figure S8: UMAPs of representative cardiomyocyte, neuronal cell and smooth muscle cell markers expressed in aCMs (3 humans).

Figure S9. UMAPs colored by myosin isoforms and troponins for human1 and human2 aCMs.

Figure S10. Slow skeletal troponin T (ssTnT) and cardiac troponin T (cTnT) expressed in aCMs (3 humans).

Figure S11. Abundance rank of representative cardiomyocyte marker proteins in aCMs and iCMs.

Figure S12. ClueGO enrichment comparisons between cluster0 vs. cluster1 in aCMs (human1 and human2) and iCMs cluster 0 vs. cluster 2.

Figure S13. Dataset completeness, mis-cleavage rate, and percent coefficients of variation (%CV) for protein and peptide quantification in iPSC differentiation dataset and its associated HeLa QC samples.

Figure S14. CellenONE isolation parameters for elongation, intensity, and cell circularity comparisons between iCMs and aCMs.

Figure S15. Extracted ion chromatograms of peptides detected in neuronal proteins in iCMs and aCMs.

**Supplementary Tables**

Table S1: ClueGo pathway and cellular component of day21 iCM cluster 0 and iCM cluster 2.

Table S2: Human subjects donor demographics

Table S3: Filtered and log transformed protein quantitative data reported by gene name and protein accession number in day21 iCMs dataset. Number of unique peptides assigned to each protein and protein sequence % coverage in iPSC-CMs dataset.

Table S4: Filtered and log transformed protein quantitative data reported by gene name and protein accession number in aCMs dataset. Number of unique peptides assigned to each protein and protein sequence % coverage in aCMs dataset.

**Contents uploaded onto the online repository at MassIVE**

All the DIA-NN search output text files with a “report.” prefixes relevant to adult human CMs dataset:

- report.gg_matrix.tsv
- report.log.txt
- report.pg_matrix.tsv
- report.pr_matrix.tsv
- report.stats.tsv
- report.tsv
- lib.tsv
- lib.tsv.speclib

All the DIA-NN search output text files with “report1.” prefixes are relevant to iPSCs-CMs differentiation dataset:

- report1.gg_matrix.tsv
- report1.log.txt
- report1.pg_matrix.tsv
- report1.pr_matrix.tsv
- report1.stats.tsv
- report1.tsv
- report.lib1.tsv
- report-lib1.tsv.speclib

**Supplementary Figures**


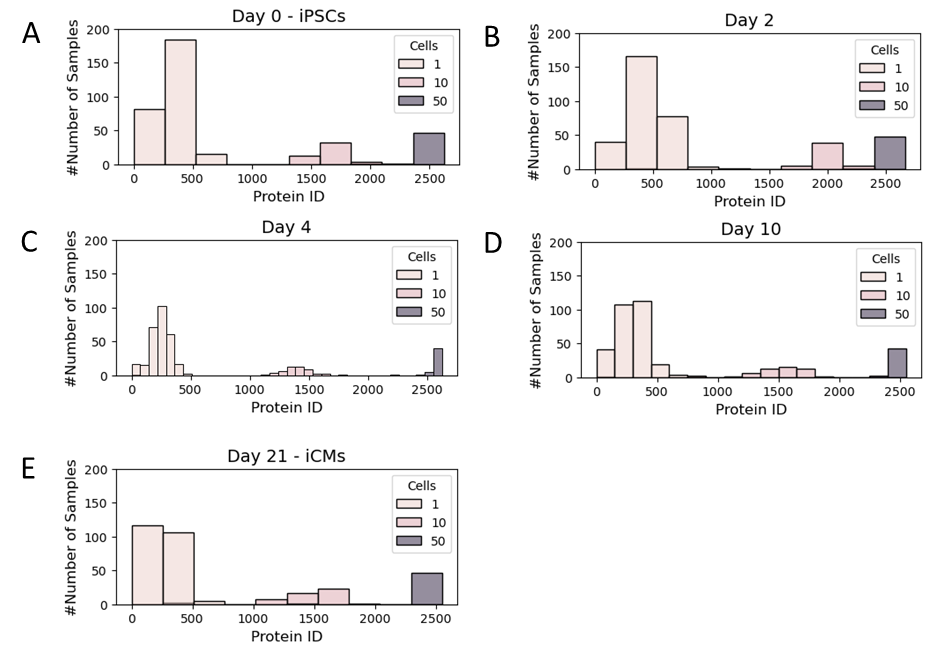


Figure S1. Protein coverage of the single cells (light pink), 10 cells (dark pink), and 50 cells (gray) from each differentiation stage: Day 0 – iPSCs (A), Day 2 (B), Day 4 (C), Day 10 (D), and Day 21 iCMs (E).


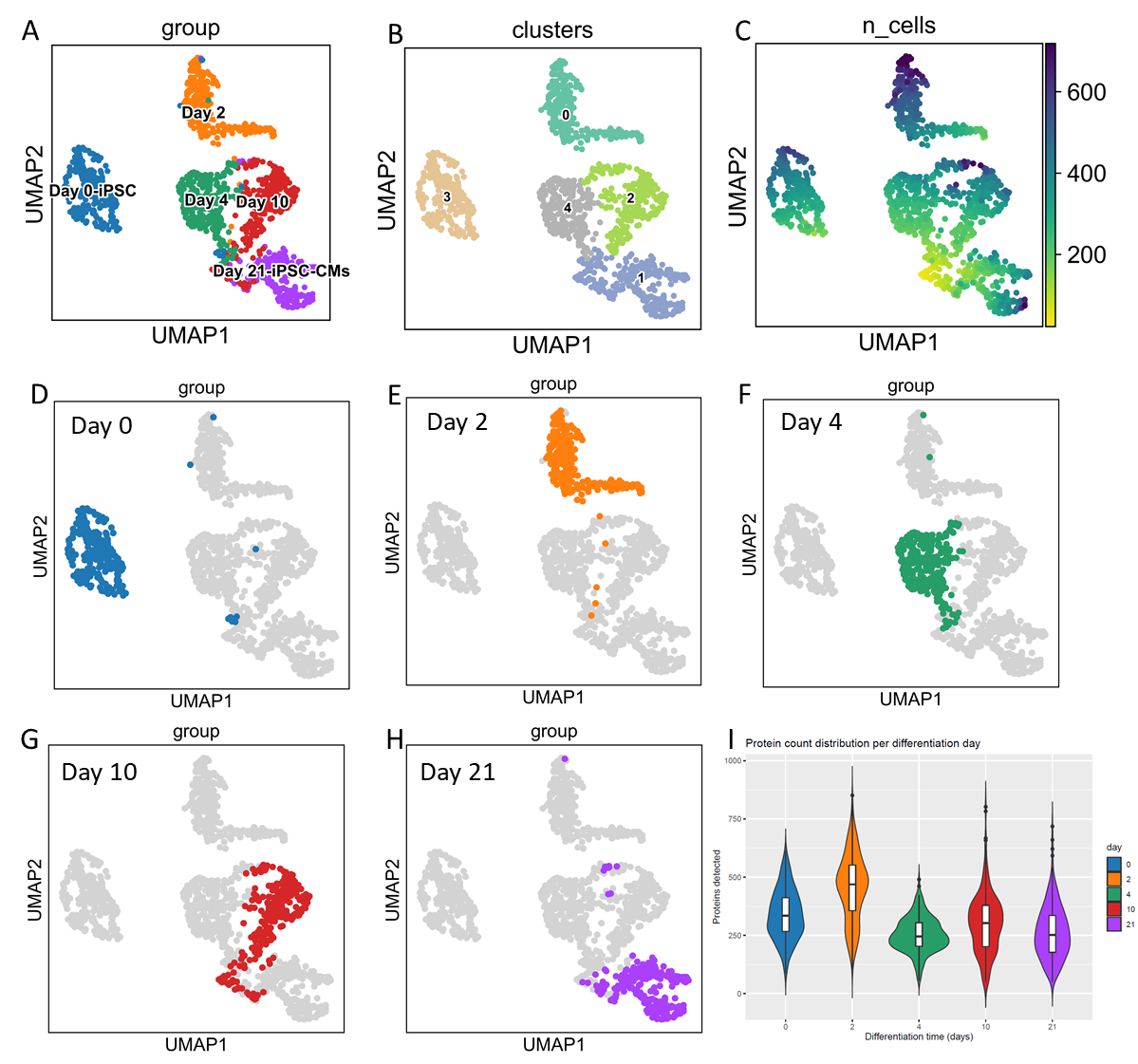


Figure S2: Distinct proteomic profiles at each differentiation timepoint. A: UMAP of individual single cell proteomes colored by the differentiation stages (n = 1326 cells). B: UMAPs of 5 Leiden clusters of the single cell proteomes. C. UMAP of each single cell colored with the number of proteins detected. D-H: UMAPs highlighting each differentiation stages separately. I: Protein count distribution per differentiation stage. Day 0-iPSC: dark blue, Day 2: orange, Day 4: dark green, Day 10: red, and Day 21-iCMs: purple.


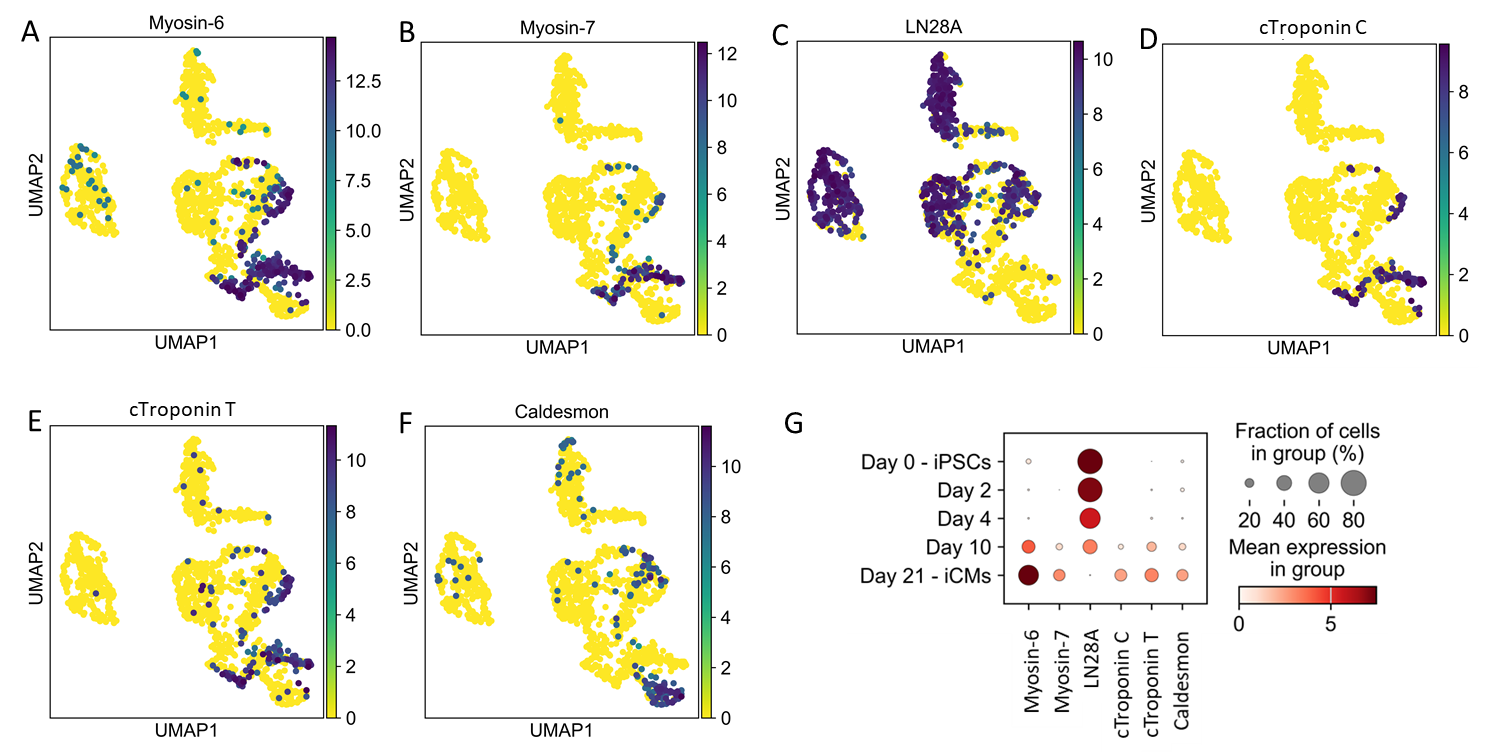


Figure S3. Cardiomyocyte marker expression increase during differentiation. A-H: individual cardiomyocyte marker expression at the single cell level at different differentiation stages visualized using UMAPs. Myosin-6 (A), Myosin-7 (B), LN28A (C), cTroponin C (D), cTroponin T (E), and smooth muscle cell marker Caldesmon (F). G. Dot plot showing the expression of the proteins per differentiation stage. The color represents the mean expression within each of the differentiation stage and the dot size indicates the fraction of cells in the categories expressing the protein.


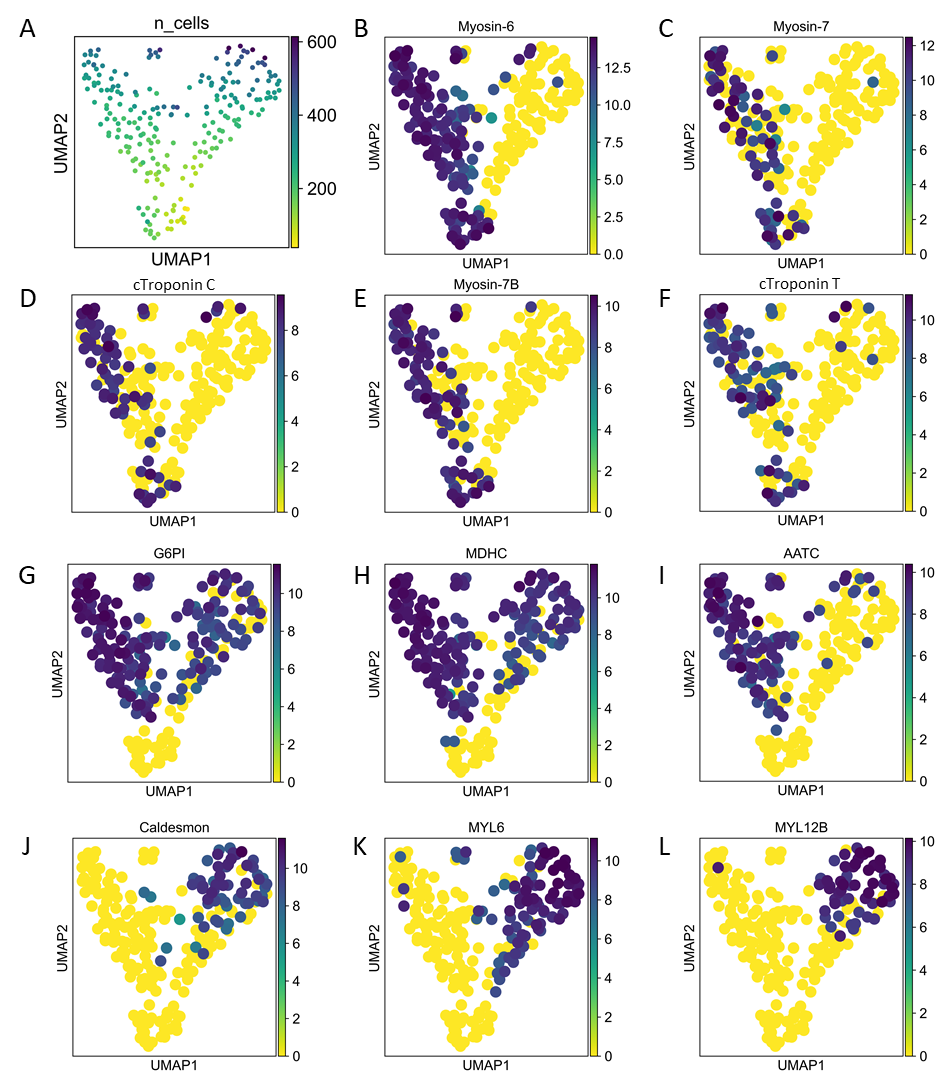


Figure S4: UMAPs of representative cardiomyocyte, metabolic and smooth muscle cell markers in Day-21 iCM cells. Number of proteins in each iCM markers (A), Myosin-6 (B), Myosin-7 (C), cTroponin C (D), Myosin-7B (E), cTroponin T (F), G6PI (G), MDHC: Malate dehydrogenase, cytoplasmic (H), AATC: Aspartate aminotransferase, cytoplasmic (I), smooth muscle cell markers Caldesmon (J), MYL6 (K), and MYL12B (L).


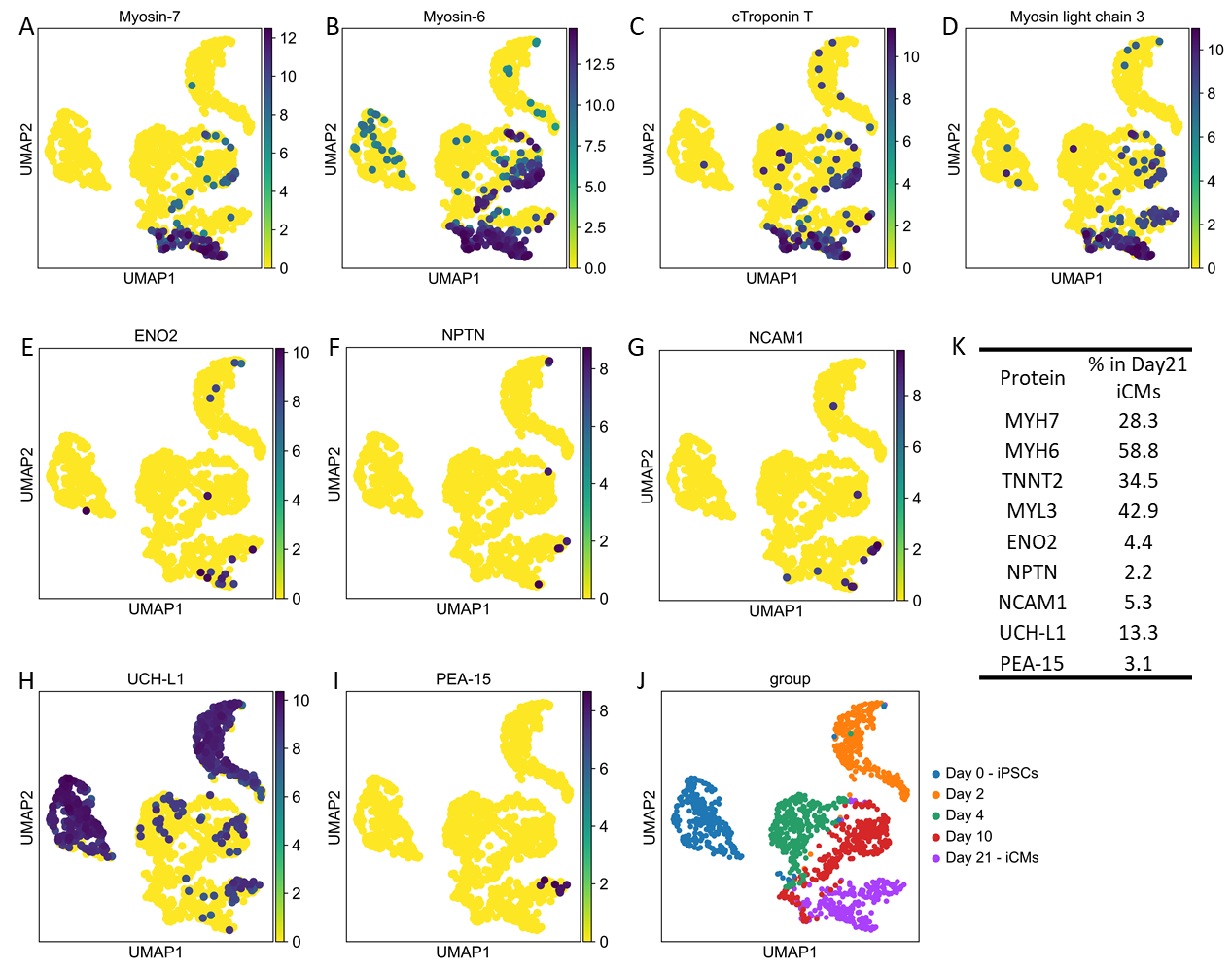


Figure S5: UMAPs of representative cardiomyocyte or neuronal cell marker expression progression through iCM differentiation. Myosin-7 (A), Myosin-6 (B), Cardiac Troponin C – TNNT2 (C), Myosin light chain 3 – MYL3 (D), Enolase 2 – ENO2 (E), Neuroplastin - NPTN (F), Neural cell adhesion molecule 1 – NCAM1 (G), Ubiquitin carboxyl-terminal hydrolase isozyme L1 – UCH-L1 (H), Astrocytic phosphoprotein PEA-15 (I), individual cell proteome colored by differentiation timepoint (J), and the list of representative proteins with their percentages of coverage in Day21 iCMs (K).


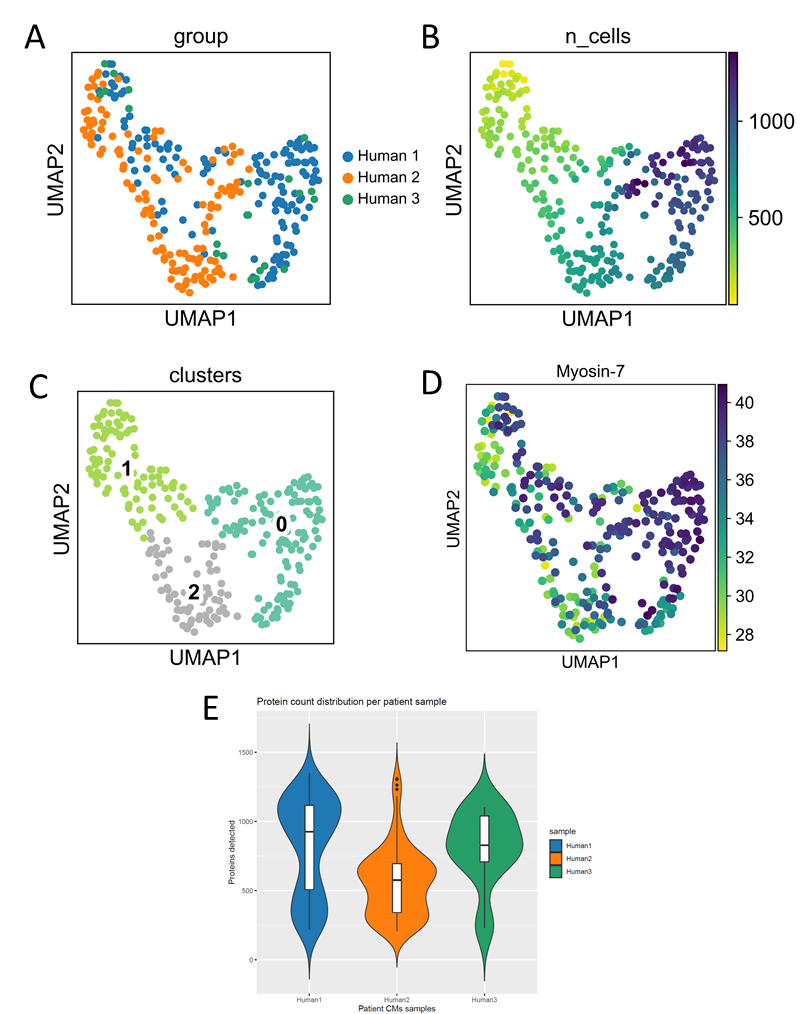


Figure S6. A. UMAP plot of aCMs from 3 human subjects combined colored by individual humans. B. UMAP plot showing the numbers of proteins identified in each aCM. C. Leiden algorithm suggested three clusters in the aCMs. D. UMAP plot of myosin-7 expression in each aCMs from three human subjects. E. Protein count distribution per patient sample.


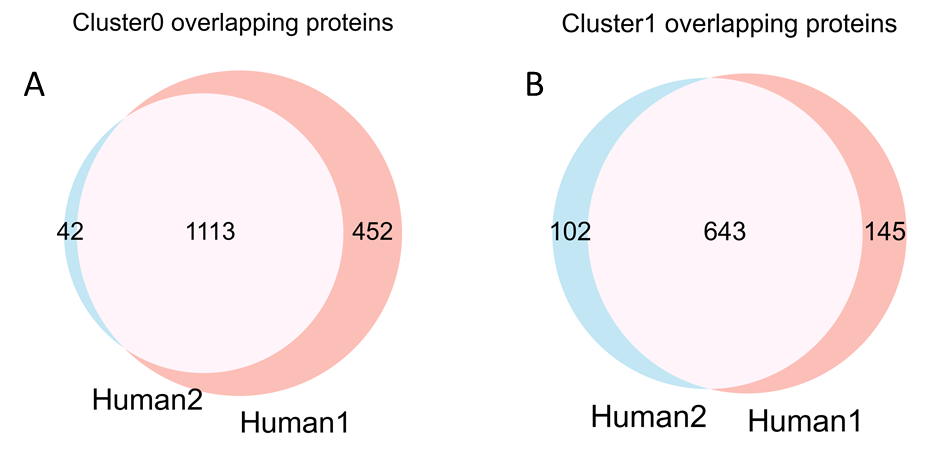


Figure S7. Proteome overlaps between cluster 0 cells (A) and cluster 1 cells (B) in human1 and human2.


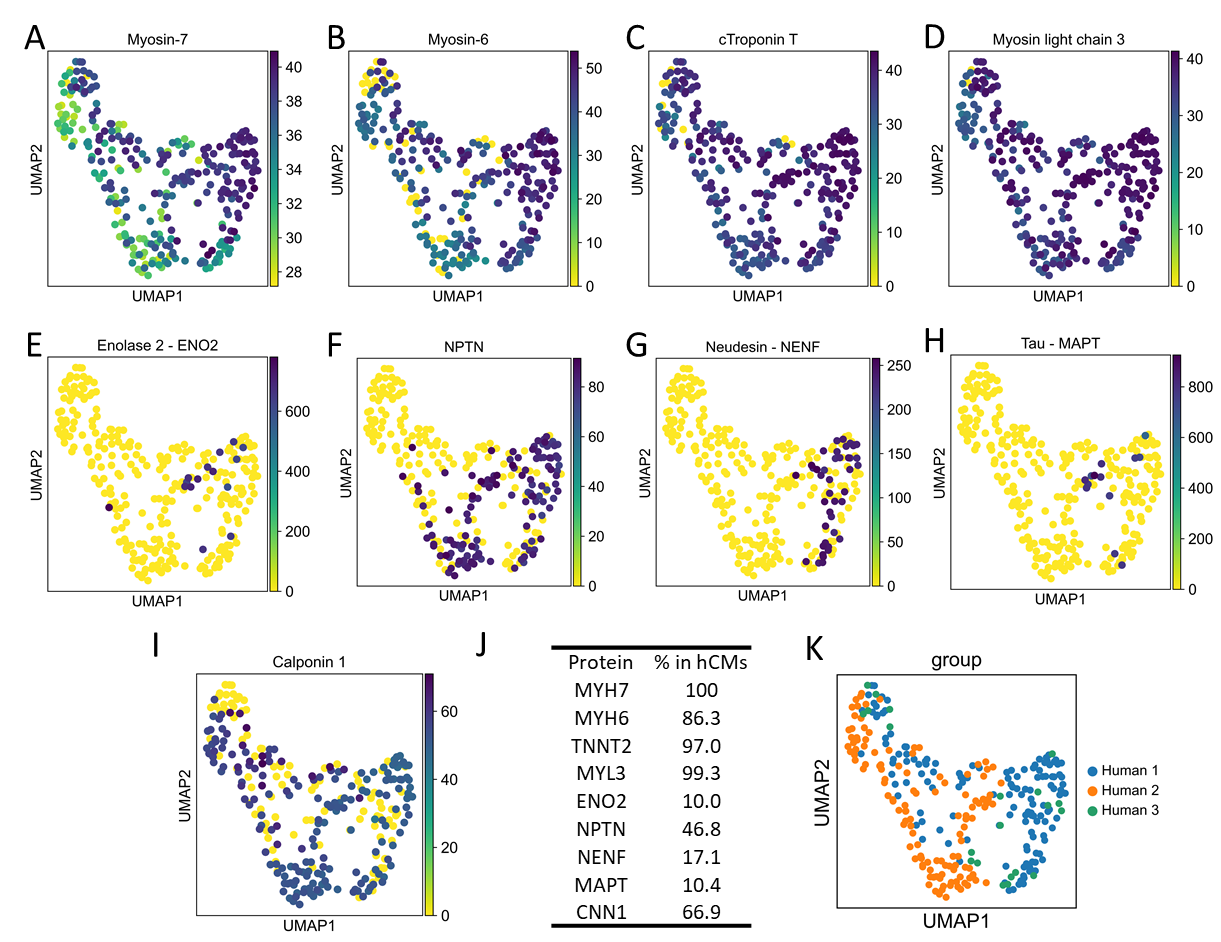


Figure S8: UMAPs of representative cardiomyocyte (A-D) or neuronal cell markers (E-H), or smooth muscle cell markers (I) expressed in aCMs (3 humans). Myosin-7 (A), Myosin-6 (B), Cardiac Troponin C – TNNT2 (C), Myosin light chain 3 – MYL3 (D), Enolase 2 – ENO2 (E), Neuroplastin - NPTN (F), Neudesin – NENF (G), Tau – MAPT (H), Calponin 1 (I); the list of representative proteins with their percentages of coverage in aCMs (J), and aCM proteomes colored by human (K).


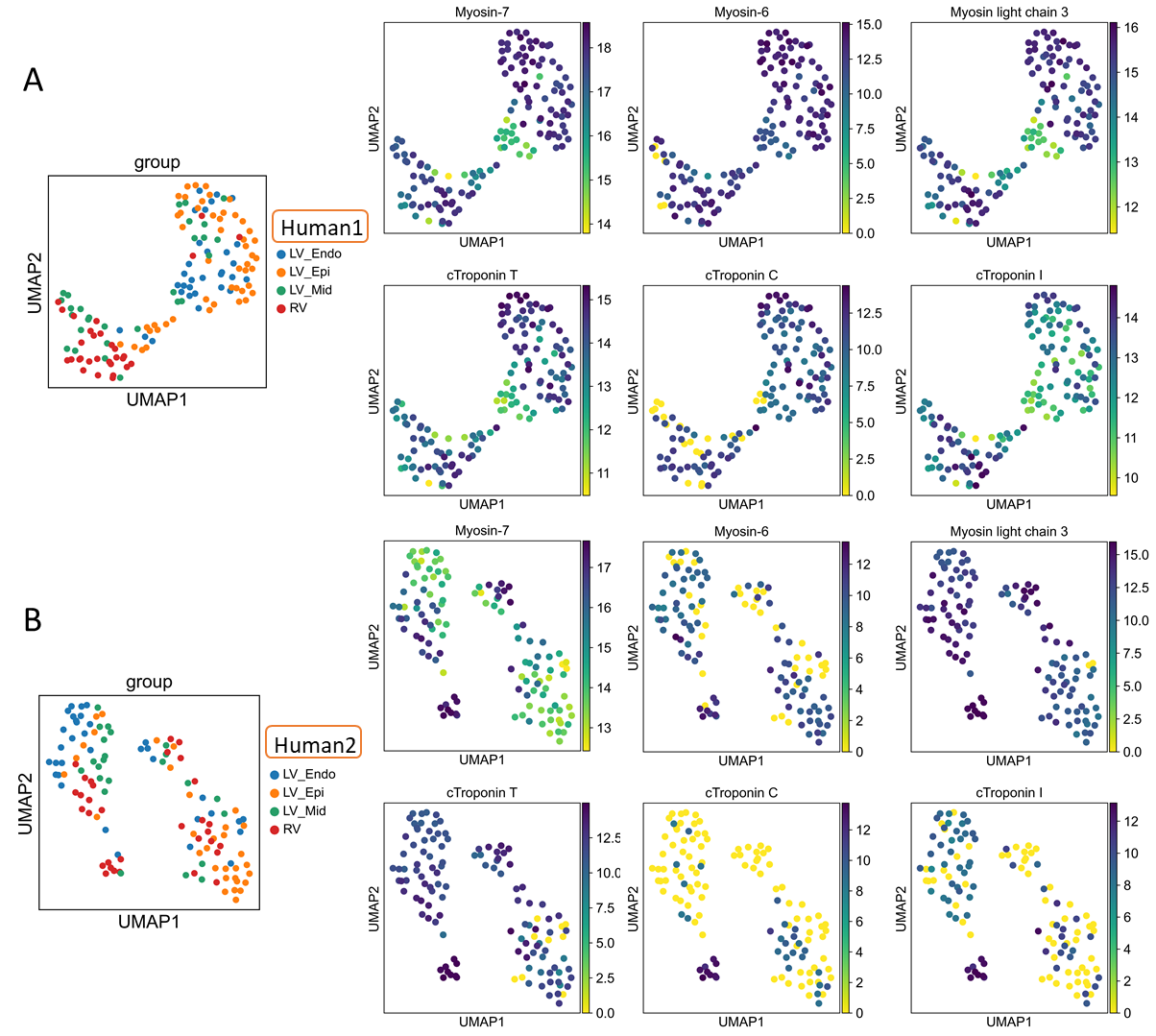


Figure S9. UMAPs colored by myosin isoforms and troponins for human1 (A) and human2 (B) aCMs.


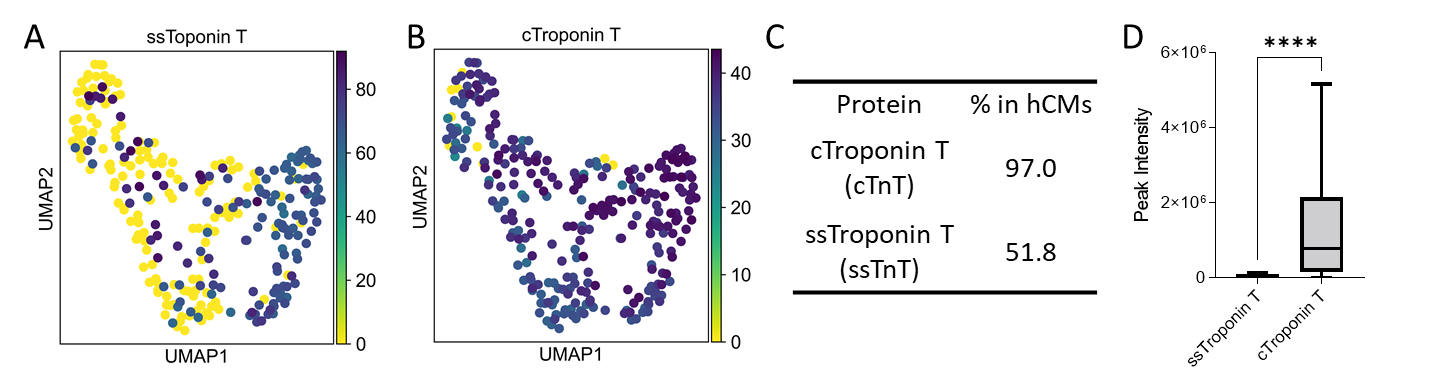


Figure S10: A. UMAP of slow skeletal troponin T (ssTnT) expressed in aCMs (3 humans). B. UMAP of cardiac Troponin T – cTnT. C. The percentages of cardiac troponin T and slow skeletal troponin T coverages in aCMs D. The MS peak intensity comparison between cardiac troponin T and slow skeletal troponin T. p****<0.0001 by t-test (n_cTroponin T_ = 290 cells; n_ssTroponin T_ = 155 cells).


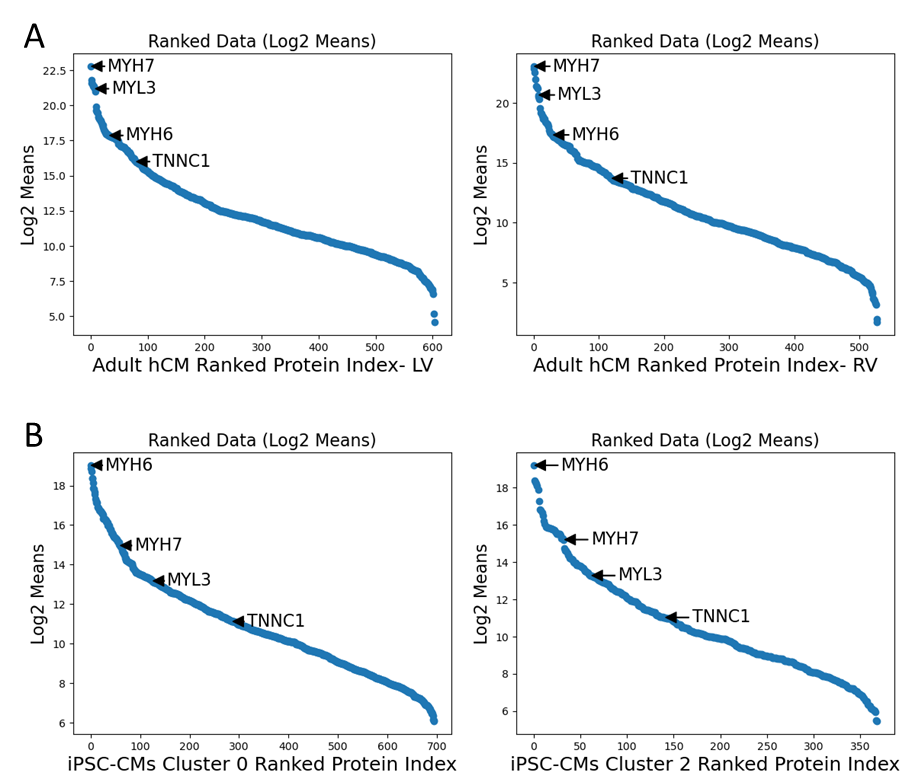


Figure S11. Abundance rank of representative cardiomyocyte marker proteins in aCMs and iCMs. Log intensity (y-axis) vs rank (left – least abundant left to right – most abundant) curves for aCMs (A, LV and RV) and iCMs (B cluster 0 and cluster 2).


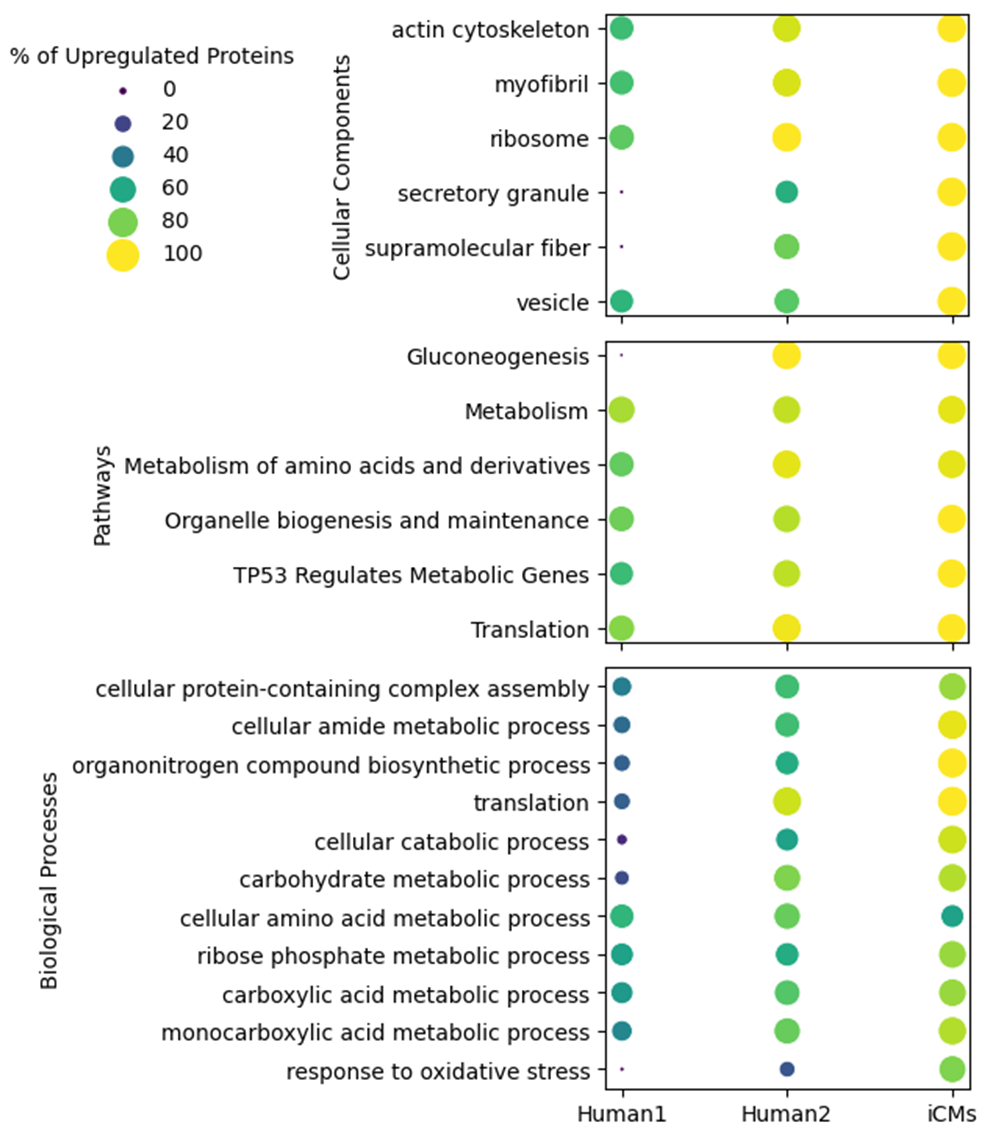


Figure S12. ClueGO enrichment comparisons between cluster0 vs. cluster1 cells show similarities between aCMs (human1 and human2) and iCMs cluster 0 vs. cluster 2 in cellular components, pathways, and biological processes.


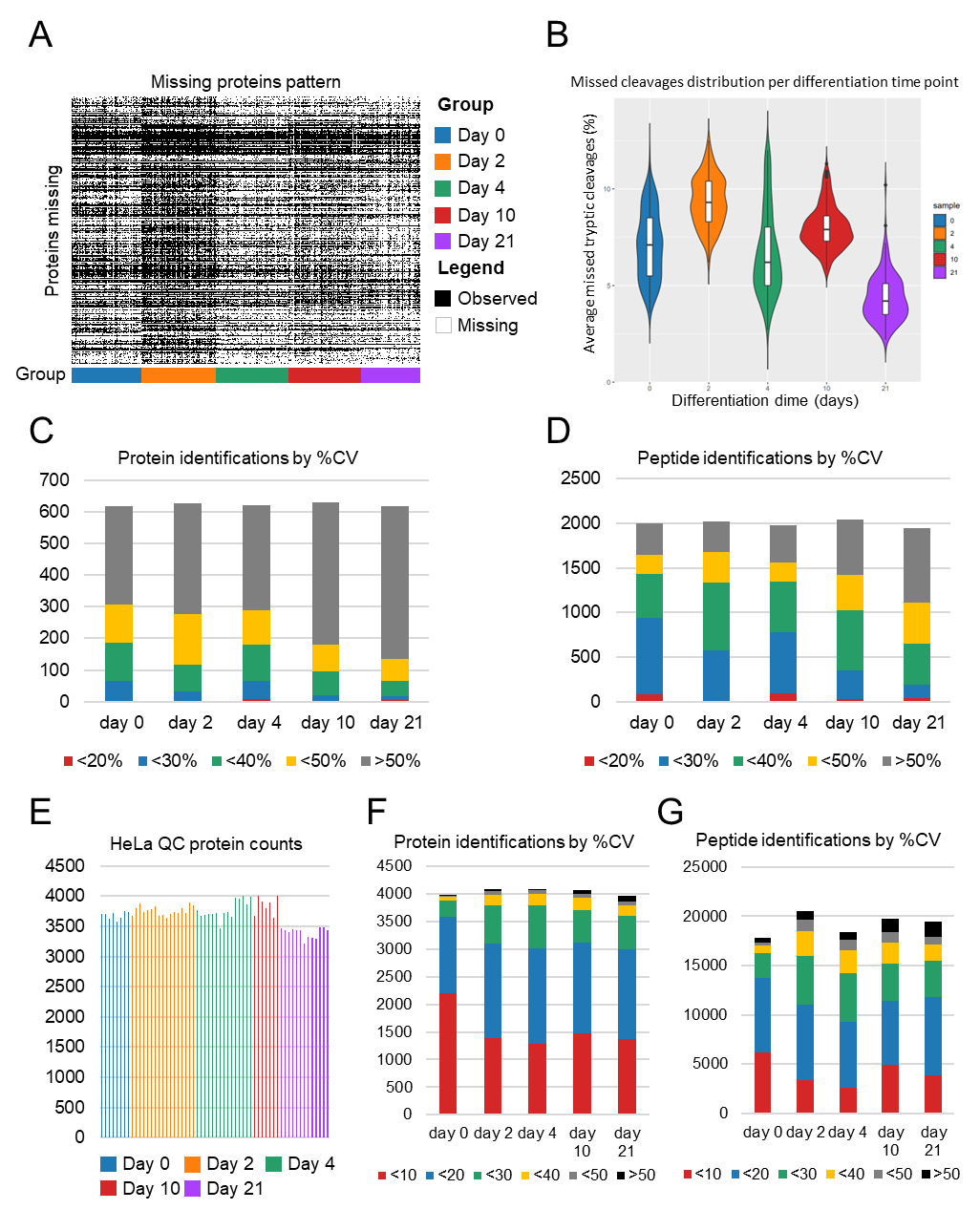


Figure S13. Dataset completeness, mis-cleavage rate and percent coefficients of variation (%CV) for protein and peptide quantification in iPSC differentiation dataset and its associated HeLa QC samples. Protein missingness plot in iPSC differentiation time course showing observed (black) and missing (white) data points across all samples (A); Missed tryptic cleavages distribution was ranging between 3-10% across all experimental groups (B); Protein and peptide identification counts in iPSC differentiation dataset represented by %CV of quantitation (C-D); HeLa QC samples protein counts (E), Protein and peptide identification counts in associated HeLa QC samples represented by %CV of quantitation (F-G).


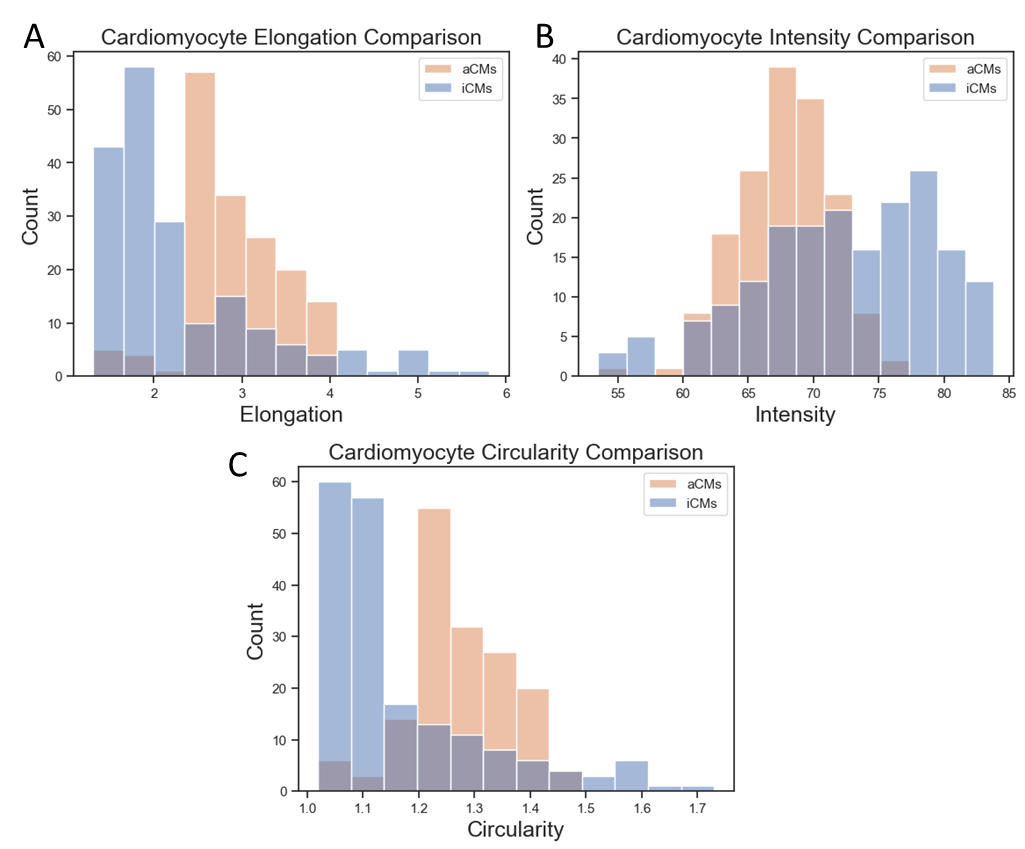


Figure S14. CellenONE isolation parameters for elongation (A), intensity (B), and cell circularity (C) comparisons between iCMs and aCMs.


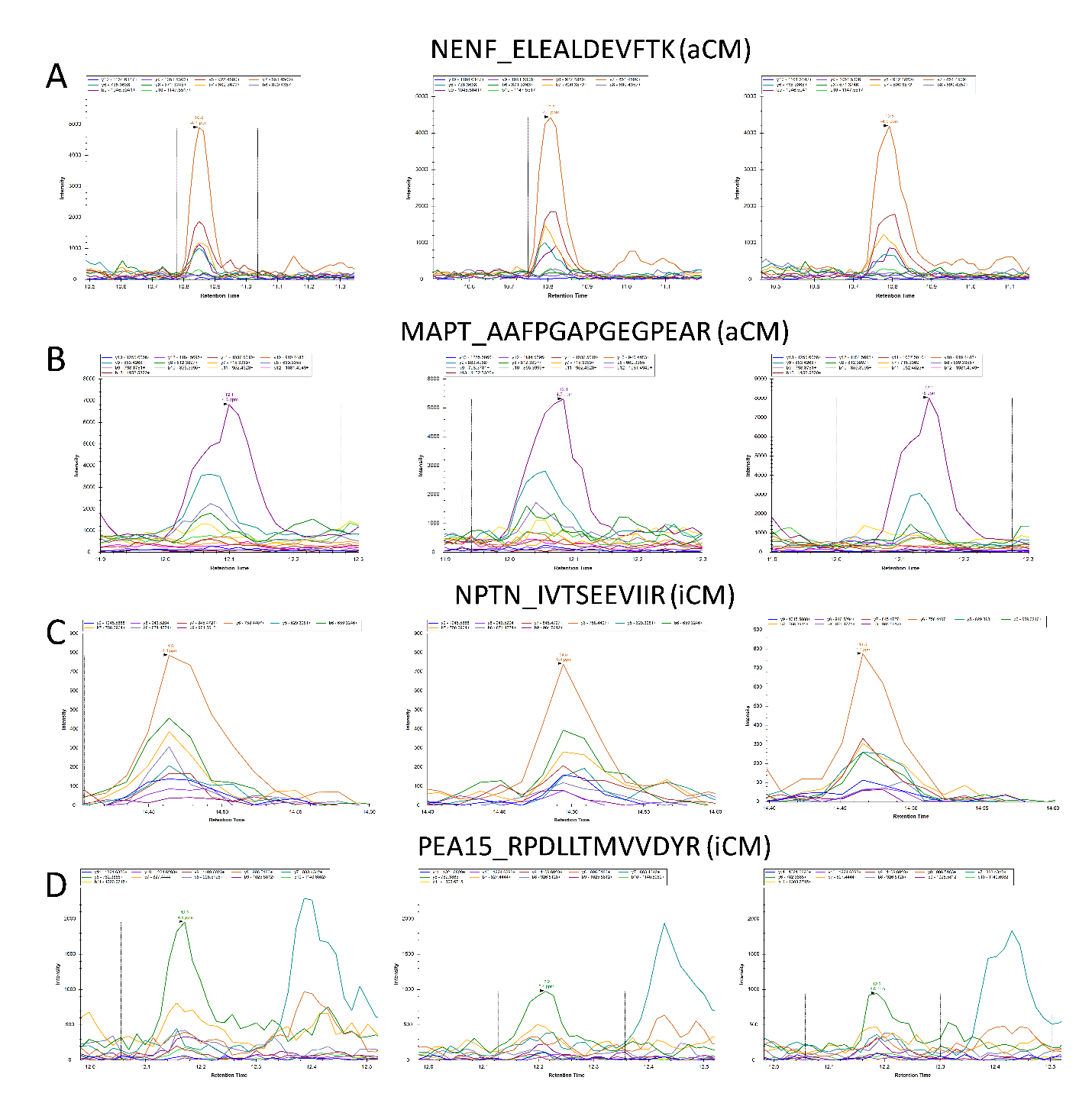


Figure S15. Extracted ion chromatograms of peptides detected in neuronal proteins in iCMs and aCMs. Presence of neuronal peptides acquired on the TimTOF SCP were visualized in Skyline (A) Quantified transitions of NENF_ELEALDEVFTK in aCMs. (B) Quantified transitions of MAPT_AAFPGAPGEGPEAR in aCMs. (C) Quantified transitions of NPTN_IVTSEEVIIR in iCMs. (D) Quantified transitions of PEA15_RPDLLTMVVDYR in iCMs.

**Supplementary Tables**


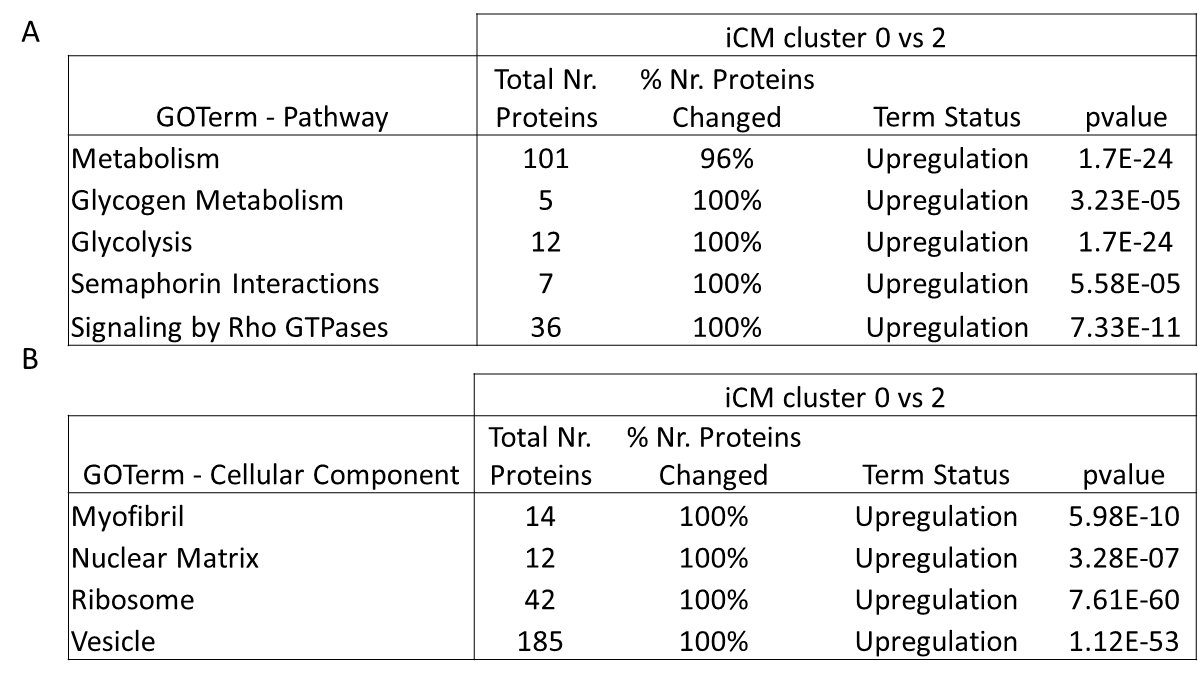


Table S1: ClueGo pathway and cellular component of day21 iCM cluster 0 and iCM cluster 2. Total 268 differentially expressed proteins between the two clusters were analyzed using ClueGO, including 264 proteins significantly upregulated in cluster 0, and 4 proteins significantly upregulated in cluster 2.


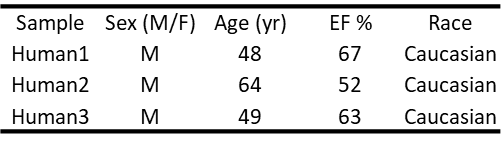


Table S2: Human subjects donor demographics.
